# GABAergic neurons are a key cell type in a *Drosophila* model of PARK14/*PLA2G6*-associated neurodegeneration

**DOI:** 10.3389/fnins.2025.1534243

**Published:** 2025-12-01

**Authors:** Noah S. Meimoun, Shimshon Benji, William Z. Besharim, Yair Y. Cantor, Eitan S. Carroll, Benjamin I. Coplin, Moshe B. Davidovics, Michael Gerber, Philip M. Hirschprung, Ephraim I. Jacobson, Aryeh L. Levenbrown, David T. Levitt, Adam Levy, Yehuda Z. Mazin, Avishye D. Moskowitz, Jeremy I. Purow, Amiel Rimberg, Jacob E. Rothstein, Eli Yaakov Saks, Rafael Saperstein, Yosef Y. Scher, Yisroel D. Schwarcz, Matthew Silver, Yitzchak F. Stein, Yisrael Y. Wiener, Josefa Steinhauer

**Affiliations:** Department of Biology, Yeshiva University, New York, NY, United States

**Keywords:** *PLA2G6*, *PLA2G6-*associated neurodegeneration, *PARK14*, Parkinson’s disease, locomotor decline

## Abstract

The causes of sporadic Parkinson’s Disease (PD) are still unclear, despite its prevalence. By contrast, inherited parkinsonian disorders have a clear genetic basis and have been studied intensively in laboratory organisms, including *Drosophila melanogaster.* Because inherited parkinsonian disorders clinically resemble sporadic PD, it has been suggested that they may share an underlying etiology. Loss of function mutations in the gene *PLA2G6* give rise to inherited neurodegenerative diseases including autosomal recessive early onset parkinsonism (PARK14). Using RNAi to deplete the *Drosophila PLA2G6* homolog *iPLA2-VIA*, we asked whether subsets of neurons, identified by their neurotransmitter usage, were more susceptible to loss of this gene. To model movement disorders connected with *PLA2G6-*associated neurodegeneration, we used the well-established climbing assay. Our results demonstrated that loss of *iPLA2-VIA* in GABAergic neurons alone strongly affected locomotor ability in aged flies, similar to pan-neuronal knockdown. Depletion of *iPLA2-VIA* in both dopaminergic and serotonergic neurons weakly affected locomotor ability as well. Depletion in other neuronal subsets did not disrupt locomotion. Furthermore, reintroducing wild-type *iPLA2-VIA* into only the dopaminergic neurons of fly knockouts improved climbing performance slightly, while reintroduction into GABAergic neurons rescued climbing performance strikingly, as well as lifespan. Although much research on this gene has focused on the dopaminergic neurons, whose degeneration leads to clinical parkinsonism, our results highlight the importance of GABAergic neurons to *PLA2G6-*associated neurodegeneration. Because sporadic PD is not thought to impact most GABAergic neurons directly, our data support the idea that sporadic PD and PARK14 have distinct etiologies despite overlapping clinical presentations.

## Introduction

Parkinson’s disease (PD) is one of the most prevalent and fastest growing neurodegenerative disorders worldwide (Dorsey and Bloem, 2018; Tanner and Ostrem, 2024). The disease is characterized and diagnosed by its four cardinal motor symptoms: bradykinesia, resting tremor, muscular rigidity, and gait and/or postural disturbances (Jankovic, 2008), which arise from death of the dopamine producing neurons in the substantia nigra pars compacta (SNpc; Dickson, 2012). Available treatments are palliative and typically involve strategies to increase dopamine levels in the brain (Kalia and Lang, 2015; Hayes, 2019; Politis et al., 2012). A major goal for the 21st century is to develop treatments that can slow, halt, or reverse disease progression (Dorsey and Bloem, 2018; Vijiaratnam et al., 2021).

The cytopathological hallmarks of PD are Lewy bodies (LBs) and Lewy neurites (LNs), intraneuronal inclusions that are characterized by the presence of the presynaptic protein *α*-synuclein (α-syn; Choong and Mochizuki, 2022; Spillantini et al., 1997). Detailed post-mortem analysis has revealed the presence of α-syn^+^ LBs and LNs in numerous regions of the central and peripheral nervous system beyond the dopaminergic SNpc neurons (Braak et al., 2003; Beach et al., 2009; Braak et al., 2006), consistent with the fact that PD patients usually present with additional non-motor symptoms, including sleep disruption, mood swings, sensory loss (especially smell), dementia, and disturbances in autonomic nervous system function (Jankovic, 2008; Blesa et al., 2022; Horsager et al., 2022). Still, Lewy pathology appears to be limited to select neuronal populations, the mechanistic basis of whose vulnerability is not fully elucidated (Surmeier et al., 2017; Braak et al., 2003; Walsh and Selkoe, 2016). The fact that *α*-syn is an intrinsically disordered protein that can form oligomers and amyloid fibrils *in vitro* and *in vivo* has led to current models suggesting that PD is caused by *α*-syn aggregation and seeding of LBs and LNs, which then cause neuronal death in the SNpc and elsewhere (Iwai et al., 1995; Weinreb et al., 1996; Spillantini et al., 1998; Goldberg and Lansbury, 2000; Wong and Krainc, 2017; Rietdijk et al., 2017; Jucker and Walker, 2013; Mahul-Mellier et al., 2020). Furthermore, disease progression has been suggested to result from spreading of Lewy pathology between neuron types and brain regions (Braak et al., 2003; Klingelhoefer and Reichmann, 2015). The mechanisms that lead to *α*-syn aggregation, Lewy pathology, spreading, and neuronal death are still poorly understood (Surmeier et al., 2017; Burke et al., 2008; Jellinger, 2009).

Although PD etiology is obscure, a genetic contribution is suggested by the existence of familial parkinsonian disorders, several of which are clinically indistinguishable from sporadic PD (Guadagnolo et al., 2021; Trinh and Farrer, 2013; Miki et al., 2017). Genetic analysis of these disorders has led to the identification of more than 20 causative “*PARK*” genes (Guadagnolo et al., 2021; Ayajuddin et al., 2018; Deng et al., 2018). The fact that human variants in the α-syn gene *SNCA (PARK1)*, or multiplications of the *SNCA* locus, underpin autosomal dominant parkinsonism supports a common etiology between sporadic PD and inherited parkinsonian disorders, and there is mounting evidence that other *PARK* gene variants also contribute to sporadic PD (Jankovic, 2008; Kim et al., 2024; Nalls et al., 2011; Do et al., 2011). Thus, it is expected that investigating the *PARK* genes will be relevant to understanding the development and progression of sporadic PD as well as inherited parkinsonism (Miki et al., 2017). The presence of *PARK* gene orthologs in other animal species has permitted development of numerous laboratory parkinsonism models, including in *Drosophila melanogaster*, which have yielded important insights into various cellular and molecular aspects of parkinsonian diseases (Ayajuddin et al., 2018; Hewitt and Whitworth, 2017). Nonetheless, the precise relationship between the various inherited parkinsonian disorders and sporadic PD still is unclear.

Mutations in the group 6 phospholipase A2 gene *PLA2G6* were identified in 2009 as the underlying cause of a familial inherited dystonia-parkinsonism disorder (Paisan-Ruiz et al., 2009; Sina et al., 2009). Since then, this gene also has been associated with autosomal recessive early onset parkinsonism and has been designated PARK14 (Paisán-Ruiz et al., 2010; Shi et al., 2011; Guo et al., 2018; Chu et al., 2020). Five groups have independently generated *Drosophila* models of *PLA2G6*-associated neurodegeneration with loss of function mutations in the fly ortholog *iPLA2-VIA* (Mori et al., 2019; Banerjee et al., 2021; Lin et al., 2018; Kinghorn et al., 2015; Iliadi et al., 2018). Loss of *iPLA2-VIA* leads to a constellation of neurodegenerative signs and symptoms, including several that have been documented in other *Drosophila* parkinsonism models such as loss of the dopaminergic neurons (Mori et al., 2019) and age-dependent loss of locomotor ability, observable in negative geotaxis climbing assays (Mori et al., 2019; Banerjee et al., 2021; Kinghorn et al., 2015; Iliadi et al., 2018). Furthermore, loss of *iPLA2-VIA* in *Drosophila* has been proposed to exacerbate *α*-syn aggregation, apparently linking this gene to development of sporadic PD (Mori et al., 2019).

Neurodegeneration in *iPLA2-VIA* mutants is not limited to the dopaminergic neurons. Prior reports have documented cell death throughout the brain (Iliadi et al., 2018) and degeneration of photoreceptor neurons in the retinas (Lin et al., 2018) of *Drosophila iPLA2-VIA* mutants. In order to better understand the extent of neuronal sensitivity to loss of *iPLA2-VIA,* we used RNAi to knock down the gene in subsets of neurons, defined by their neurotransmitter usage (Deng et al., 2019). To monitor the effect of our manipulations, we used the climbing assay, which is a well-established behavioral read-out of neurodegeneration (Barone and Bohmann, 2013). After confirming prior reports that pan-neuronal knockdown of *iPLA2-VIA* can phenocopy the climbing defect of the knockout mutant (Banerjee et al., 2021; Iliadi et al., 2018), we demonstrated that knockdown in GABAergic neurons alone can fully phenocopy the strong age-dependent climbing defect. By contrast, knockdown in octopaminergic-tyraminergic, cholinergic, or glutamatergic neurons did not affect climbing ability up to 30 days of age. Knockdown in dopaminergic and serotonergic neurons produced a small age-dependent climbing defect that was much weaker than that produced by knockdown in GABAergic neurons. Moreover, reintroducing wild-type *iPLA2-VIA* into GABAergic neurons of the mutant strongly rescued defective climbing, while reintroduction in dopaminergic neurons rescued weakly. Reintroducing wild-type *iPLA2-VIA* into GABAergic neurons also rescued the lifespan of the mutants. Altogether, our data indicate that GABAergic neurons are an important site of *iPLA2-VIA* neuroprotective activity, that severe locomotor defects arise when the gene is lost from these cells, and that restoring wild-type *iPLA2-VIA* in these cells can slow age-dependent loss of locomotor ability and death. This is consistent with clinical reports of patients suffering from *PLA2G6-*associated neurodegeneration (PLAN), who consistently show high penetrance degeneration of GABAergic cerebellar tissue (Guo et al., 2018; Gregory et al., 2008; Salih et al., 2013). However, this stands in contrast to sporadic PD, in which predominantly GABAergic regions such as the cerebellum and globus pallidus are largely spared from degeneration (Dickson, 2012; Braak et al., 2003).

## Materials and methods

### Fly stocks and culture

*Drosophila* were raised on standard media. Experimental crosses and F1 cohorts were kept at 26 °C under 12 h L:D cycle. Fly strains were *w^1118^ (BDSC_5905), HMS01544 (BDSC_36129),yw (BDSC_6599), yv; attP2 (BDSC 36303), elav-GAL4 (BDSC_458), ple-GAL4 (BDSC_8848), Vesicular GABA Transporter (VGAT)-GAL4 (BDSC_58980), Dopa decarboxylase (Ddc)-GAL4 (BDSC_7010), Tryptophan hydroxylase (Trh)-GAL4 (BDSC_38388), Tyrosine decarboxylase 2 (Tdc2)-GAL4 (BDSC_9313),* C*holine Acetyltransferase (ChAT)-GAL4 (BDSC_56500)*, and *Vesicular glutamate transporter (VGluT)*-*GAL4 (BDSC_26160)* from Bloomington *Drosophila* Stock Center. We used FlyBase to find information on the stocks listed above. The *iPLA2-VIA* knockout mutant ∆23 and the *UAS*-*iPLA2-VIA-PB* line were described previously (Banerjee et al., 2021).

### Climbing assays

F1 flies were collected every 2–3 days, males were grouped into cohorts of up to 12 flies, and they were transferred to new food every 5 days during aging. Climbing assays were conducted on each group at 10, 20, and 30 days from eclosion and were performed at room temperature as described in Banerjee et al. (2021). Briefly, each fly cohort was transferred to a fresh food vial and a clean empty vial was placed on top of the food vial. Flies were tapped to the bottom and given 20 s to climb 6 cm. Each assay consisted of five climbing trials. Each fly earned one point for successfully climbing to or past the 6 cm mark within 20 s. The total number of points for the group was divided by the number of flies in the group to determine the climbing index. Climbing indices were assessed for normality using Shapiro–Wilk test, and statistical comparisons were made using two-way ANOVA and Tukey’s post-hoc comparisons, performed in R. Graphs were made in R with ggplot2. Graphs show average climbing index per condition, error bars are SEM. At least 8 groups were sampled per condition. Genotypes and sample sizes are given in Table 1.

**Table 1:** Genotypes and samples sizes.

	10 days		20 days		30 days	
Genotype	Groups	Flies	Groups	Flies	Groups	Flies
Knockdown climbing						
*VGAT-GAL4/+*	8	71	11	90	11	90
*VGAT-GAL4/+; UAS-iPLA2-VIA-RNAi/+*	8	79	9	86	10	91
*Ddc-GAL4/+*	15	133	15	128	14	97
*Ddc-GAL4/+; UAS-iPLA2-VIA-RNAi/+*	15	133	15	128	15	114
*elav-GAL4/Y*	20	193	17	164	16	153
*elav-GAL4/Y; UAS-iPLA2-VIA-RNAi/+*	17	186	17	182	16	170
*ple-GAL4/+*	10	99	10	98	10	98
*ple-GAL4/UAS-iPLA2-VIA-RNAi*	10	95	9	84	9	73
*Trh-GAL4/+*	10	102	10	93	10	97
*Trh-GAL4/UAS-iPLA2-VIA-RNAi*	12	127	12	115	12	112
*Tdc2-GAL4/+*	16	125	16	110	12	88
*Tdc2-GAL4/+; UAS-iPLA2-VIA-RNAi/+*	15	136	15	129	15	114
*ChAT-GAL4/+*	12	114	12	105	11	96
*ChAT-GAL4/+; UAS-iPLA2-VIA-RNAi/+*	12	119	11	104	10	96
*VGlut-GAL4/+*	10	85	9	63	9	60
*VGlut-GAL4/+; UAS-iPLA2-VIA-RNAi/+*	17	147	16	127	16	122

	15 days		20 days	
	Groups	Flies	Groups	Flies
Climbing rescue				
*VGAT-GAL4/+; iPLA2-VIA^∆23/∆23^*	12	84	11	76
*VGAT-GAL4/UAS-iPLA2-VIA-PB; iPLA2-VIA^∆23/∆23^*	13	85	13	83
*ple-GAL4, iPLA2-VIA^∆23^/+, iPLA2-VIA^∆23^*	14	113	14	111
*UAS-iPLA2-VIA-PB/+; ple-GAL4, iPLA2-VIA^∆23^/+, iPLA2-VIA^∆23^*	14	113	14	102
Flies				
Lifespan rescue				
*VGAT-GAL4/+*	109			
*VGAT-GAL4/+; iPLA2-VIA^∆23/∆23^*	66			
*VGAT-GAL4/UAS-iPLA2-VIA-PB; iPLA2-VIA^∆23/∆23^*	104			

### Lifespan assay

F1 flies were collected every 2–3 days without gas. After 2–3 days, F1 males of the appropriate genotype were separated over light gas and grouped into cohorts of up to 15 flies per vial. Thereafter, F1 cohorts were passed without gas to fresh vials every 2–3 days. Censors (escapers) and deaths were recorded at each pass. Adapted from Delventhal et al. (2022) and Piper and Partridge (2016). Kaplan–Meier curves were generated in R. Statistical comparison by log-rank test. Genotypes and sample sizes are given in Table 1.

## Results

### Knocking down *iPLA2-VIA* in GABAergic or dopaminergic and serotonergic neurons leads to age-dependent climbing defects

*iPLA2-VIA* loss of function leads to reduced climbing ability after 20 days of age post-eclosion (Banerjee et al., 2021; Kinghorn et al., 2015; Iliadi et al., 2018). To determine which neurons are sensitive to the loss of *iPLA2-VIA*, we knocked down the gene in discrete neuronal subsets according to their neurotransmitter production. We used well-characterized *GAL4* drivers that target distinct neuronal populations in the adult fly nervous system using regulatory sequences from genes involved in neurotransmitter synthesis or transport (Deng et al., 2019; Chen et al., 2013). Climbing assays were conducted at 10, 20, and 30 days post-eclosion.

**Figure 1:**
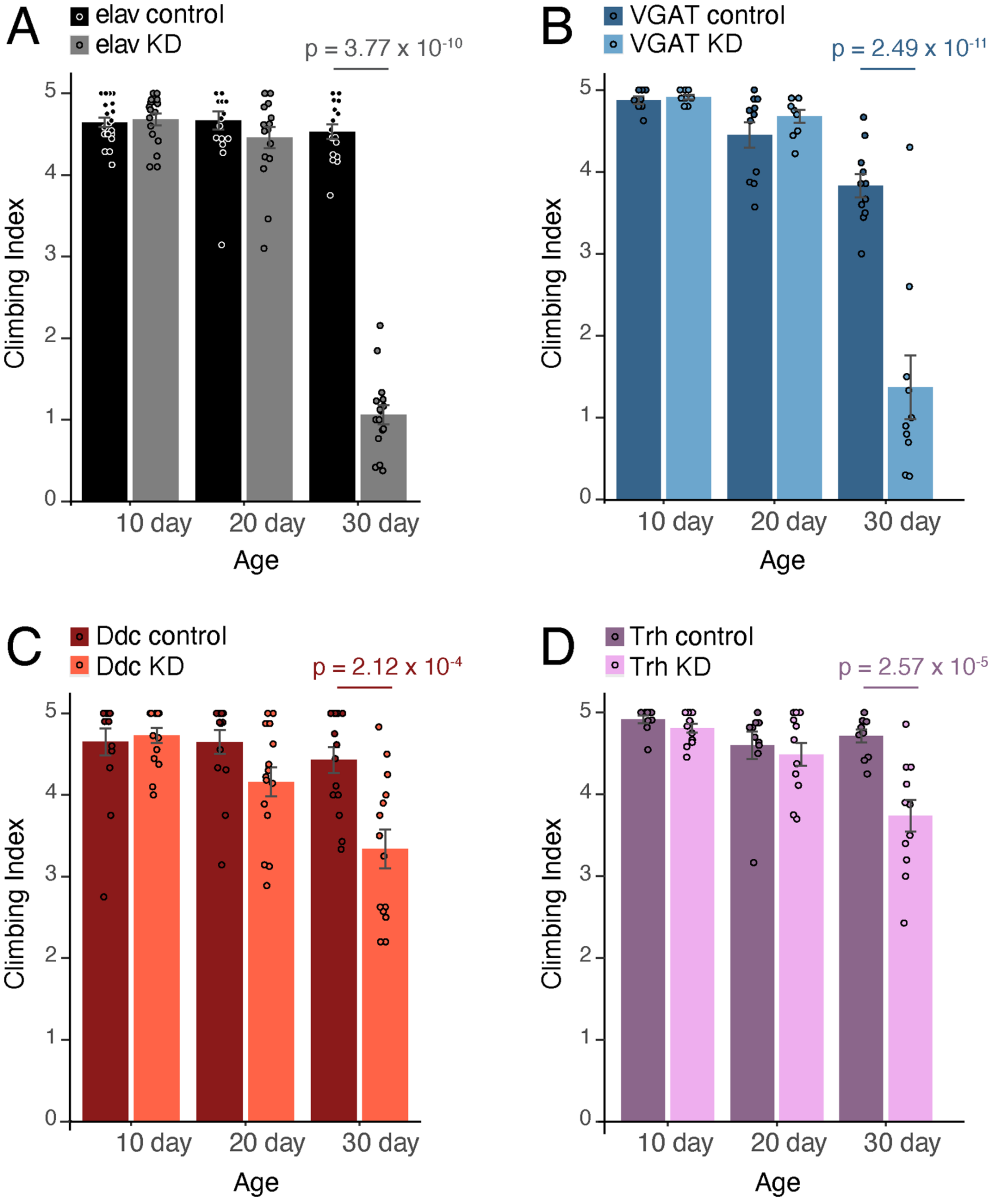
Knocking down *iPLA2-VIA* in GABAergic or dopaminergic and serotonergic neurons leads to age-dependent climbing defects. **(A)** Climbing ability was dramatically reduced in flies expressing *iPLA2-VIA* RNAi with the pan-neuronal driver *elav-GAL4* (gray) at day 30, while climbing ability was maintained in age-matched control flies expressing *elav-GAL4* alone (black). *p* = 3.77 × 10^−10^ for 30 day knockdown (KD) compared to 30 day control. **(B)** Climbing ability was dramatically reduced in flies expressing *iPLA2-VIA* RNAi with the GABAergic driver *VGAT-GAL4* (light blue) at day 30 compared to age-matched control flies carrying *VGAT-GAL4* alone (dark blue), phenocopying the effect seen with pan-neuronal knockdown. *p* = 2.49 × 10^−11^ for 30 day KD compared to 30 day control. Note that 30 day *VGAT-GAL4* control flies had slightly reduced climbing ability compared to 10 day *VGAT-GAL4* control flies, *p* = 0.00727, but neither age point was significantly different from 20 day *VGAT-GAL4* control flies. **(C)** Flies expressing *iPLA2-VIA* RNAi in dopaminergic and serotonergic neurons with *Ddc-GAL4* showed reduced climbing ability at day 30 (light red), while age-matched control flies carrying *Ddc-GAL4* alone did not lose climbing ability (dark red). *p* = 2.12 × 10^−4^ for 30 day KD compared to 30 day control. **(D)** Flies expressing *iPLA2-VIA* RNAi in serotonergic neurons with *Trh-GAL4* had reduced climbing ability at day 30 (light purple), while age-matched control flies carrying *Trh-GAL4* alone retained climbing ability (dark purple). *p* = 2.57 × 10^−5^ for 30 day KD compared to 30 day control.

We confirmed that pan-neuronal knockdown of *iPLA2-VIA* using the *elav-GAL4* driver produced a strong climbing defect at 30 days of age, as demonstrated previously (Figure 1A, gray bars; Banerjee et al., 2021; Kinghorn et al., 2015; Iliadi et al., 2018). This effect was age-dependent, as it is for the *iPLA2-VIA* knockout mutant. To knock down *iPLA2-VIA* in only GABAergic neurons, we used the *Vesicular GABA Transporter (VGAT)-GAL4* driver (Fei et al., 2010), which resulted in a strong age-dependent climbing defect that phenocopied the effect observed with pan-neuronal knockdown (Figure 1B, light blue bars).

Reports from multiple model systems have documented the sensitivity of dopaminergic neurons to *iPLA2-VIA* loss of function (Mori et al., 2019; Sanchez et al., 2018; Chiu et al., 2019). We therefore used the *Dopa decarboxylase (Ddc)-GAL4* driver to knock down *iPLA2-VIA* (Li et al., 2000), which caused a small but significant age-dependent loss of climbing ability as expected (Figure 1C, light red bars). Because *Ddc-GAL4* is expressed in both dopaminergic and serotonergic neurons, we established two separate sets of crosses using either *pale (ple)-GAL4* to target *iPLA2-VIA* in dopaminergic neurons only (Friggi-Grelin et al., 2003) or the *Tryptophan hydroxylase (Trh)-GAL4* driver for serotonergic neurons only (Alekseyenko et al., 2010). Knockdown in serotonergic neurons caused a similar age-dependent loss of climbing ability (Figure 1D, light purple bars), but surprisingly, knockdown in dopaminergic neurons alone did not reduce climbing ability (Supplementary Figure S1A). Thus, our data suggest that loss of *iPLA2-VIA* in serotonergic neurons, and likely also dopaminergic neurons, leads to age-dependent climbing defects.

### Knocking down *iPLA2-VIA* in octopaminergic and tyraminergic, cholinergic, or glutamatergic neurons does not lead to age-dependent climbing defects

In the same study, we tested an additional three neuronal subsets. We knocked down *iPLA2-VIA* in octopaminergic and tyraminergic neurons using *Tyrosine decarboxylase 2 (Tdc2)-GAL4* (Cole et al., 2005), in cholinergic neurons using *Choline Acetyltransferase (ChAT)-GAL4* (Salvaterra and Kitamoto, 2001), or in glutamatergic neurons using *Vesicular Glutamate Transporter (VGluT)*-*GAL4* (Daniels et al., 2008). None of these manipulations caused climbing defects (Supplementary Figures S1B–D).

### Restoring *iPLA2-VIA* in GABAergic or dopaminergic neurons rescues climbing defects

**Figure 2:**
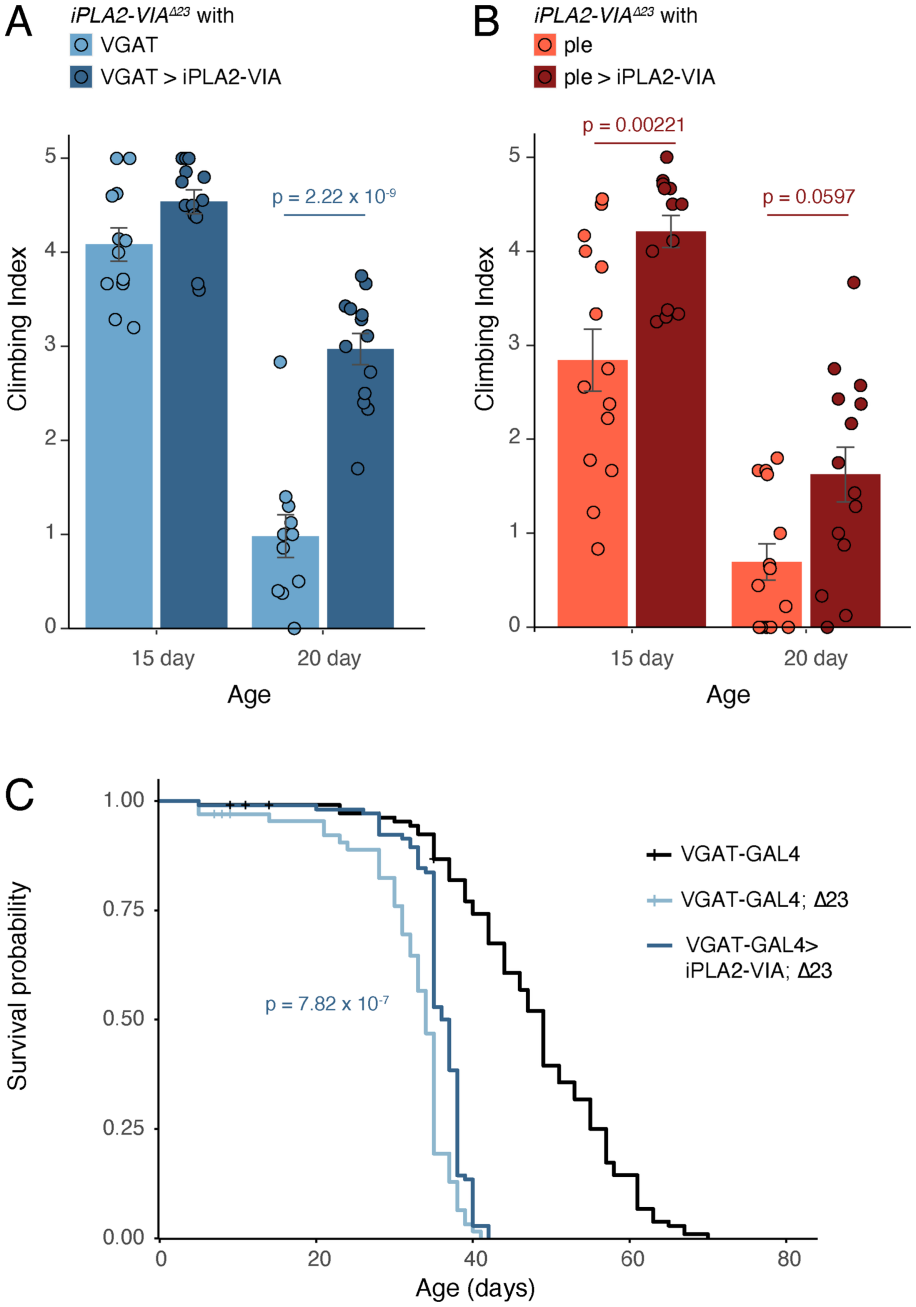
*iPLA2-VIA* knockout mutants are rescued by expression of wild-type *iPLA2-VIA* in GABAergic or dopaminergic neurons. **(A)** *iPLA2-VIA^∆23^* flies carrying the GABAergic driver *VGAT-GAL4* showed dramatically reduced climbing ability at 20 days of age at 26 °C (light blue). This was rescued strongly by expression of *UAS-iPLA2-VIA-PB* (dark blue), *p* = 2.22 × 10^−9^. For 15 day rescue versus 15 day control, *p* = 0.260 (not significant). **(B)** *iPLA2-VIA^∆23^* flies carrying the dopaminergic driver *ple-GAL4* showed reduced climbing ability at 20 days of age at 26 °C (light red), which was rescued weakly by expression of *UAS-iPLA2-VIA-PB* (dark red), *p* = 0.0597. *iPLA2-VIA^∆23^* flies carrying *ple-GAL4* also showed slightly reduced climbing ability at 15 days of age, which was improved by expression of *UAS-iPLA2-VIA-PB*, *p* = 0.00221. **(C)** *iPLA2-VIA^∆23^* mutant flies (light blue, carrying *VGAT-GAL4*) had severely reduced lifespans compared to control flies (black, *VGAT-GAL4/+*) at 26 °C. *iPLA2-VIA^∆23^* mutants expressing *UAS-iPLA2-VIA-PB* with *VGAT-GAL4* (dark blue) showed significantly improved survival, *p* = 7.82 × 10^−7^ for rescued flies compared to mutant flies by log-rank test. Age in days post-eclosion. Censors indicated by “|”.

The above experiments showed that *iPLA2-VIA* is necessary in GABAergic neurons, and to a lesser extent in dopaminergic and serotonergic neurons, to maintain normal climbing ability with age. To assess whether *iPLA2-VIA* is sufficient in these neurons, we used *GAL4* to express a *UAS*-driven wild-type *iPLA2-VIA-PB* transgene in the *iPLA2-VIA^∆23^* mutant, as described previously (Banerjee et al., 2021). Note that in wild-type flies with *iPLA2-VIA* knockdown, climbing defects were not evident until after 20 days of age. By contrast, *iPLA2-VIA^∆23^* mutant flies showed strong climbing defects at 20 days of age at 26 °C. This discrepancy likely is due to the pleiotropic effect of losing *iPLA2-VIA* from multiple vulnerable tissue types in the mutant (Banerjee et al., 2021). Nevertheless, despite the requirement for *iPLA2-VIA* in multiple tissue types, wild-type *iPLA2-VIA* expressed in the GABAergic neurons of mutant flies using the *VGAT-GAL4* driver demonstrated a striking rescue of climbing ability at 20 days of age (Figure 2A, dark blue bars). Expressing wild-type *iPLA2-VIA* in dopaminergic neurons using the *ple-GAL4* driver also improved deficient climbing of the mutant at both 15 and 20 days of age, albeit to a weaker extent than expression in GABAergic neurons (Figure 2B, dark red bars). Together, our knockdown and rescue results show that *iPLA2-VIA* is necessary and sufficient in GABAergic neurons for protection from age-dependent loss of climbing ability.

### Restoring *iPLA2-VIA* in GABAergic neurons rescues lifespan

Loss of function *iPLA2-VIA* mutants have severely shortened lifespans, a common symptom of neurodegeneration (Lin et al., 2018; Kinghorn et al., 2015; Iliadi et al., 2018). Therefore, as an additional test for the importance of GABAergic neurons, we assessed whether restoring wild-type *iPLA2-VIA-PB* in GABAergic neurons only could rescue the lifespan of *iPLA2-VIA^∆23^* mutants. Notably, rescued mutant flies carrying *VGAT-GAL4* and the wild-type *iPLA2-VIA-PB* transgene had significantly improved survival compared to mutant flies carrying just *VGAT-GAL4* (Figure 2C), affirming that GABAergic neurons are a critical cell type in PLAN.

## Discussion

In this study, we examined the effect of knocking down *iPLA2-VIA* in different neuron types, classified by their neurotransmitter usage, on age-dependent climbing ability. Our results identify GABAergic neurons as a key neuronal population in *PLA2G6-*associated neurodegeneration. Depletion of *iPLA2-VIA* expression in GABAergic neurons using RNAi led to severe age-dependent climbing defects similar to those caused by depleting *iPLA2-VIA* pan-neuronally (Figures 1A,B), and restoration of a wild-type *iPLA2-VIA* allele in GABAergic neurons led to robust rescue of both climbing ability and lifespan in the *iPLA2-VIA* knockout mutant (Figures 2A,C). Age-dependent loss of climbing ability also was observed when *iPLA2-VIA* was knocked down in dopaminergic and serotonergic neurons with *Ddc-GAL4* (Figure 1C), and restoring the wild-type allele in dopaminergic neurons alone with *ple-GAL4* modestly rescued climbing in the mutant (Figure 2B). These data are consistent with previous studies indicating a requirement for *iPLA2-VIA* in dopaminergic neurons (Mori et al., 2019; Sanchez et al., 2018; Chiu et al., 2019). Furthermore, our results showing that *iPLA2-VIA* knockdown with *Trh-GAL4* reduced climbing ability suggest that serotonergic neurons are important as well (Figure 1D). Knockdown in other neuronal populations, i.e., octopaminergic and tyraminergic, cholinergic, and glutamatergic neurons, did not affect climbing (Supplementary Figures S1B–D).

### Limitations of the study

The negative geotaxis climbing assay has been a mainstay of *Drosophila* behavioral genetics for decades and has been used extensively to monitor neurodegeneration (Barone and Bohmann, 2013; Inagaki et al., 2010). Although numerous modifications to the assay have been developed over the years, the current study relied on a simple protocol, on account of its cost and equipment efficiencies, its utility for rapid assimilation by many experimenters, and its sufficiency in detecting age-dependent locomotor defects in *iPLA2-VIA* mutants (Banerjee et al., 2021). Still, subtle motor defects may have been missed in our study. It is also possible that in those conditions that failed to show an effect in our climbing assays, lower *GAL4* expression resulted in lower knockdown efficacy, although the drivers we used are well-characterized and commonly used (e.g., Deng et al., 2019; Chen et al., 2013; Howard et al., 2019). Lower *GAL4* expression likely explains the fact that knockdown in dopaminergic neurons only with *ple-GAL4* failed to affect climbing (Supplementary Figure S1A), despite much evidence that dopaminergic neurons are sensitive to loss of *iPLA2-VIA*. Higher resolution techniques, preferably with computerized video tracking and image processing, should be used in the future to improve detection of altered locomotion under *iPLA2-VIA* loss of function conditions (Aggarwal et al., 2019; Wu et al., 2019). Furthermore, our study did not account for other types of neurological defects aside from those affecting locomotion. Of note, although survival of *iPLA2-VIA* mutants was improved by reintroducing the wild-type allele into GABAergic neurons only, the lifespans of the rescued flies were still markedly attenuated compared to control flies, indicating the involvement of other cell types in overall survival (Figure 2C). Single cell RNA-seq has revealed widespread *iPLA2-VIA* expression in the fly brain, coincident with most neurotransmitters (Davie et al., 2018), and prior studies have shown cell death throughout the fly brain in *iPLA2-VIA* loss of function mutants (Iliadi et al., 2018). Because our study did not examine cell death histologically, it remains to be seen precisely which brain areas and/or neuron types succumb to cell death, and over what time frame.

### Relation to human *PLA2G6*-associated neurodegeneration

*Drosophila* and mammals share most of the major neurotransmitters, including GABA, dopamine, serotonin, acetylcholine, and glutamate, with replacement of the vertebrate epinephrine and norepinephrine with the functionally analogous octopamine and tyramine in insects (Deng et al., 2019; Deshpande et al., 2020). A wealth of studies also suggest many similar functions and behavioral outputs, including in learning and memory, circadian rhythms, reward sensing, etc. (Kasture et al., 2018). Nevertheless, the limited similarities in neuroanatomical organization between insects and vertebrates and the coexistence of multiple neuron types within each neuroanatomical region may impede direct extrapolation of findings between species. Moreover, the fact that many neurons produce multiple neurotransmitters may confound a simple classification system based on this property (Deng et al., 2019). With these caveats in mind, we turn our attention to human *PLA2G6-*associated neurodegeneration (PLAN).

PLAN was first described in 2006 as a collection of neurodegenerative disorders affecting children and young adults (i.e., infantile neuroaxonal dystrophy, atypical neuroaxonal dystrophy, and neurodegeneration with brain iron accumulation) with severe loss of motor coordination (Gregory et al., 2008; Morgan et al., 2006). Since then, loss of *PLA2G6* in human patients has been associated also with inherited parkinsonism, as well as with dystonia and ataxia (Paisan-Ruiz et al., 2009; Guo et al., 2018; Xue et al., 2023; Erro et al., 2017). Thus, while *PLA2G6* loss of function clearly causes movement disorders, it is less clear whether specific subsets of neurons are more vulnerable than others. Parkinsonism results from perturbations in the dopaminergic circuit of the basal ganglia, while perturbations in other areas, including the cerebellum, can lead to dystonia and ataxia (Dickson, 2012; Sharma, 2019). Consistently with the clinical symptoms, MRI studies of human PLAN patients have revealed abnormalities in multiple brain regions, and importantly, the most commonly affected brain region is the cerebellum, which is largely GABAergic (Gregory et al., 2008; Salih et al., 2013). Our results showing that age-dependent climbing behavior in *Drosophila* is strongly dependent on *iPLA2-VIA* in GABAergic neurons are in line with human clinical and MRI data, as well as with mouse *PLA2G6* knockouts showing loss of GABAergic Purkinje cells in the cerebellum (Zhao et al., 2011). So far, *Drosophila* studies have utilized straightforward amorphic or hypomorphic *iPLA2-VIA* conditions. It has been speculated that specific molecular lesions in the gene may result in distinct clinical symptoms, but evidence has been inconclusive (Guo et al., 2018; Xue et al., 2023; Engel et al., 2010). Furthermore, it is unclear how mutations in *PLA2G6* lead to neurodegeneration, although abnormalities in mitochondria, endolysosomal pathways, Ca^+2^ handling, ER and presynaptic membranes, and phospholipid acyl chain composition have been noted (Mori et al., 2019; Lin et al., 2018; Kinghorn et al., 2015; Chiu et al., 2019; Zhou et al., 2016; Beck et al., 2011).

### Pathologic process and relation to sporadic PD

Mutations in any of the more than 20 “*PARK”* genes can cause parkinsonism and, in some cases, mimic sporadic PD. However, it remains unclear how closely the underlying pathological sequence of events matches between each inherited parkinsonian disorder and sporadic PD. Loss of function mutations in *PLA2G6/*PARK14 have been suggested to induce cellular conditions similar to those occurring in sporadic PD, e.g., by causing lysosomal dysfunction (Lin et al., 2018) or by promoting *α*-syn aggregation (Mori et al., 2019). Indeed, dopaminergic neurons degenerate in both PLAN and sporadic PD (Miki et al., 2017; Mori et al., 2019; Sanchez et al., 2018; Chiu et al., 2019). Serotonergic neurons also succumb in sporadic PD (Politis et al., 2012; Braak et al., 2003), and although their status in PLAN is not well-described, this study suggests they are relevant to PLAN as well. However, in sporadic PD, GABAergic regions including the globus pallidus and cerebellum largely are spared from Lewy pathology and neurodegeneration (Dickson, 2012; Braak et al., 2003). By contrast, PLAN patients often experience degeneration in the cerebellum and iron accumulation in the globus pallidus (Guo et al., 2018; Gregory et al., 2008). Our experiments indicate that GABAergic neurons are extremely sensitive to loss of *iPLA2-VIA*, and that this can cause severe locomotor impairment. Together, our results along with the clinical and human pathology observations may suggest that the cytopathological stimuli in *PLA2G6-*associated neurodegeneration are different from those in sporadic PD, although they likely eventually converge on similar or identical pathways as neurodegeneration proceeds. This is consistent with recent large scale association studies that have failed to find a link between *PLA2G6* and sporadic PD (Kim et al., 2024; Tomiyama et al., 2011; Liu et al., 2020). To date, it is still unknown what determines the specific neuronal sensitivities in PD and other neurodegenerative conditions (Braak et al., 2003; Paß et al., 2021).

## Data Availability

The raw data supporting the conclusions of this article will be made available by the authors, without undue reservation.
