# Supplementary material for: GABAergic neurons are a key cell type in a Drosophila model of PARK14/PLA2G6-associated neurodegeneration
Source: Front Neurosci. 2025 Dec 1;19:1534243. doi: 10.3389/fnins.2025.1534243 (PMC12702979; doi:10.3389/fnins.2025.1534243)
Supplement: Supplementary file 1 [file Data_Sheet_1.docx]

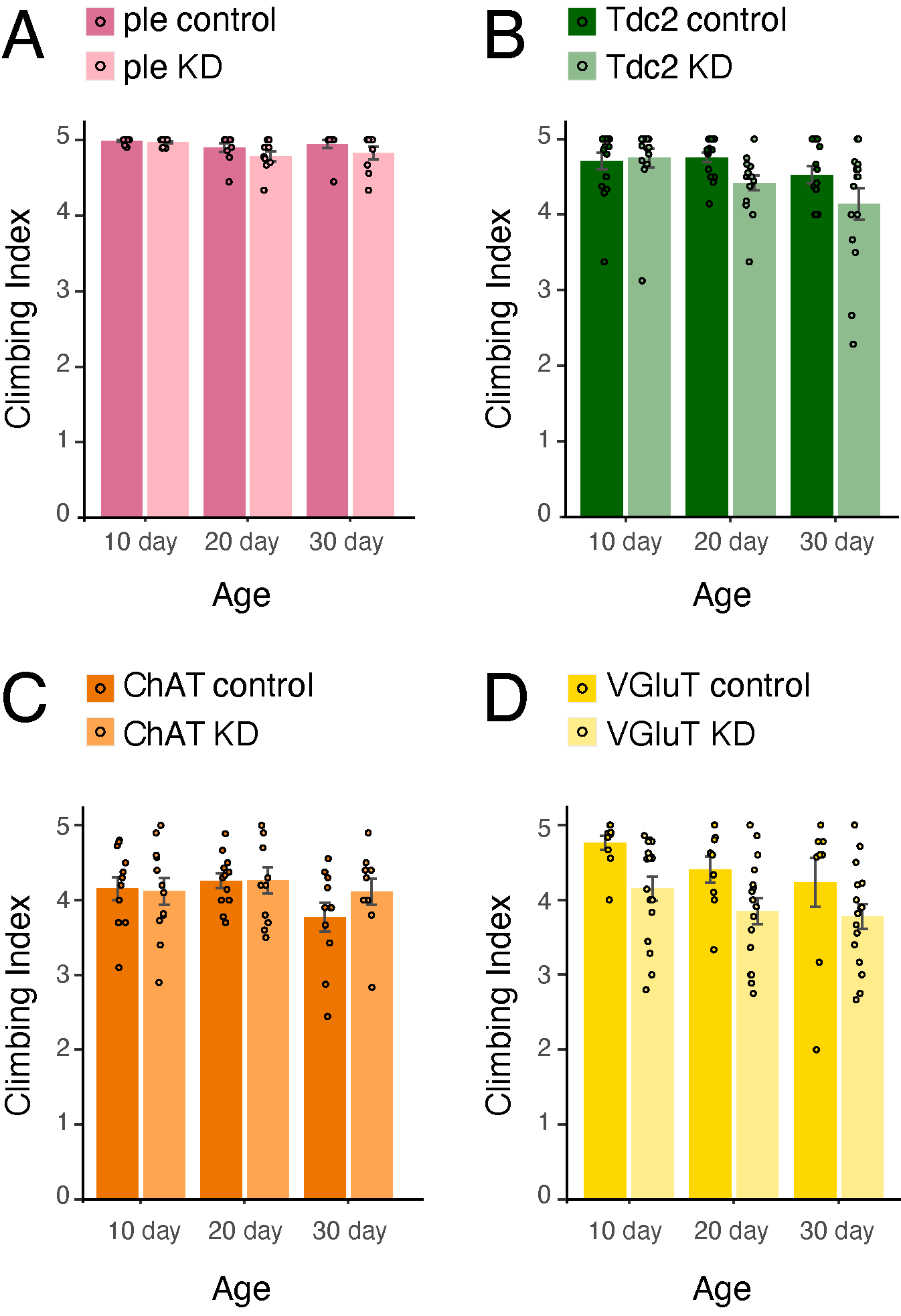


**Figure S1. Knocking down *iPLA­2­VIA* in dopaminergic neurons alone, in octopaminergic and tyraminergic neurons, in cholinergic neurons, or in glutamatergic neurons does not perturb climbing ability.** (A) Flies expressing *iPLA2­-VIA* RNAi in dopaminergic neurons only with *ple-GAL4* did not show reduced climbing ability at any age (light pink), compared to *ple-GAL4* alone (dark pink). (B) *iPLA­2­VIA* knockdown in octopaminergic and tyraminergic neurons with *Tdc2-GAL4* did not affect climbing ability (light green), compared to age-matched control flies expressing *Tdc2-GAL4* alone (dark green). (C) *iPLA2-VIA* knockdown in cholinergic neurons with *ChAT-GAL4* did not affect climbing ability (light orange), compared to age-matched control flies carrying *ChAT-GAL4* alone (dark orange). (D) *iPLA2-VIA* knockdown in glutamatergic neurons with *VGluT-GAL4* (light yellow) did not affect climbing ability, compared to control flies carrying *VGluT-GAL4* alone (dark yellow).
